# Biosurfactants for Microbubble Preparation and Application

**DOI:** 10.3390/ijms12010462

**Published:** 2011-01-17

**Authors:** Qingyi Xu, Mitsutoshi Nakajima, Zengshe Liu, Takeo Shiina

**Affiliations:** 1 Food Engineering Division, National Food Institute, 2-1-12 Kannondai, Tsukuba, Ibaraki 305-8642, Japan; 2 Graduate School of Life and Environmental Sciences, University of Tsukuba, 1-1-1 Tennoudai, Tsukuba, Ibaraki 305-8572, Japan; E-Mail: nakajima.m.fu@u.tsukuba.ac.jp; 3 NCAUR/ARS/USDA, 1815 N. University Street, Peoria, IL 61604, USA; E-Mail: Kevin.Liu@ars.usda.gov

**Keywords:** biosurfactant, microbubble, production, characterization

## Abstract

Biosurfactants can be classified by their chemical composition and their origin. This review briefly describes various classes of biosurfactants based on their origin and introduces a few of the most widely used biosurfactants. The current status and future trends in biosurfactant production are discussed, with an emphasis on those derived from plants. Following a brief introduction of the properties of microbubbles, recent progress in the application of microbubble technology to molecular imaging, wastewater treatment, and aerobic fermentation are presented. Several studies on the preparation, characterization and applications of biosurfactant-based microbubbles are reviewed.

## 1. Introduction

Surfactants are amphiphilic compounds containing both hydrophilic and lipophilic moieties. Due to their dual nature, surfactants tend to partition into the oil-air or oil-water interface to reduce the surface and interfacial tension and stabilize newly created interfaces. Surfactants can be derived from both chemically based (“chemical surfactants” or “synthetic surfactants”) and biologically based (“biosurfactants”) sources [1]. Strictly speaking, a biosurfactant is a surfactant directly derived from a natural source (*i.e.*, from a plant, animal or microorganism), but this term is often used in a broader sense to include surfactants synthesized from natural raw materials. Fatty acid esters of sugars and fatty acid esters or amides of amino acids are examples of the surfactants belonging to this category [2].

Biosurfactants offer advantages over synthetic surfactants in terms of their derivation from renewable resources, low or non-toxicity, biodegradability, excellent surface activity, possible reuse through regeneration, high specificity, and effectiveness under extreme temperature and pH conditions [1,3]. The major functions of biosurfactants include solubilization, emulsification, dispersion, wetting, foaming, and detergent capacity, as well as antimicrobial activity in some cases [3,4]. Consequently, interest in biosurfactants is continuously increasing. Biosurfactants have been used in various industries alone or blended with other biosurfactants or synthetic surfactants to offer desired performance characteristics. In the food industry, biosurfactants provide multiple functions and act as emulsifying/foaming agents, stabilizers, antioxidant agents, and antiadhesives [5–7]. Environmental and agricultural applications are major areas of biosurfactant utilization, where they play important roles in soil remediation, oil recovery, and plant pathogen elimination. Biosurfactants have also found applications in detergents, paints, coatings, cosmetics and pharmaceutics [4,5].

**Figure 1:**
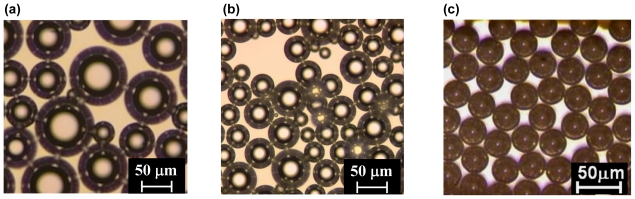
Microbubbles prepared with mechanical agitation (**a**), sonication (**b**), and microchannel emulsification (**c**).

Microbubbles are bubbles with diameters less than 100 μm. The commonly used methods for preparing microbubble dispersions include mechanical agitation, sonication, and pressurized gas-liquid mixing systems, which usually result in the formation of microbubbles with wide size distributions. Since the behavior and property of microbubbles depend strongly on their size and size distribution, the demand for preparing microbubbles with desired size and narrow size distribution is increasing. Microfluidic technologies are currently the primary methods for preparing monodisperse microbubbles. Figure 1 shows the microbubbles prepared with different generation methods.

**Figure 2:**
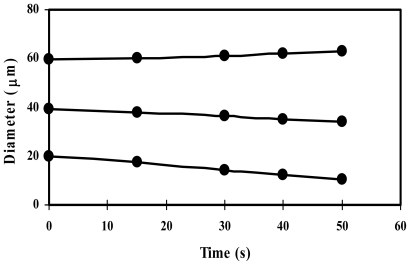
Size-dependent behavior of microbubbles.

Compared to conventional bubbles in the millimeter range, microbubbles offer novel and unique properties, such as a higher surface area-to-volume ratio, slower rising velocity in the liquid phase and higher internal pressure. An important property of microbubbles that distinguishes them from conventional bubbles is that they shrink when their size is below a critical value; the rate of shrinkage significantly increases as microbubble size decreases, due to increased internal pressure (Figure 2) [8]. Microbubble dispersions are easily handled due to their water-like viscosity [9]. As their promising properties are recognized, demand for microbubble-based products, especially microbubbles stabilized by biosurfactants, is expected to increase. However, there is a lack of available information on biosurfactant-based microbubbles and their applications. In this review, we will focus on the current knowledge of biosurfactant-based microbubbles and their applications.

## 2. Type of Biosurfactants

Biosurfactants can be classified by their chemical composition and their origin. In this review, biosurfactants are grouped into three categories of origin: microbially derived surfactants, animal-derived surfactants and plant-derived biosurfactants. Most biosurfactants are either anionic or neutral; only a few, such as those containing amine groups, are cationic [10].

### 2.1. Microbially Derived Surfactants

Microbially derived surfactants are surface-active agents synthesized by bacteria, yeasts and fungi to facilitate growth on various substrates (e.g., sugars, oils, alkanes and wastes) [11]. They vary widely in molecular weight. Low-molecular-weight biosurfactants include glycolipids, lipopeptides and phospholipids. They are effective in reducing surface and interfacial tension. High-molecular-weight biosurfactants are composed of polysaccharides, proteins, lipopolysaccharides, lipoproteins or complex mixtures of these biopolymers. They are more effective in stabilizing newly created surfaces [7,12].

The increasing interest in microbially derived surfactants is based on their wide diversity in structure and function and on their low cost, which is enabled by their production from cheaper agro-based substrates and waste materials. However, their industrial application is limited by a lack of public acceptance of producer strains, low production yields, and the high purity necessary for food, cosmetic, and pharmaceutical applications, which result in higher costs [11]. Consequently, they are mainly used in environmental applications. However, their high surface activity, biodegradability, low or non-toxicity, emulsifying and demulsifying ability, antimicrobial activity, and tolerance to wide ranges of pH, temperature and ionic strength make them promising for food, cosmetic and pharmaceutical applications [7].

### 2.2. Animal-Derived Surfactants

Typical examples of biosurfactants derived from animal sources include lecithin, gelatin, casein, wool fat, cholesterol, and wax [13]. They have a variety of uses because of their widely different chemical constitution.

Lecithin is the only truly natural low-molecular-weight surfactant available for industrial application [14]. It is a mixture of phospholipids that is a natural constituent of animals and plants. Animal-derived lecithin is usually produced from egg yolk and consists of zwitterionic phosphatidylethanolamine (PE, ~18.1%) and phosphatidylcholine (PC, ~78.7%). Purified egg lecithin is mainly used as a pharmaceutical excipient for drug delivery and intravenous nutrition [15,16].

Gelatin is a product obtained through the partial hydrolysis of collagen with dilute acid or base. The main sources of commercial gelatin are bovine skin and bones and pigskin. Increasing concern over bovine spongiform encephalopathy (BSE) has led to the development of fish gelatin alternatives. Gelatin is a high-molecular-weight polymer and had been used as a stabilizer, thickener, and texturizer in food and non-food applications [17]. It is a relatively poor protein surfactant, but its emulsifying properties can be improved by enzyme-catalyzed attachment of hydrophobic side chains [14] or by combination with other surface-active agents.

Casein is a milk protein that accounts for ~80% of milk’s total protein content. It is a heterogeneous phosphoprotein that consists of four major fractions: α_s1_ (~44%), α_s2_ (~11%), β (~32%), and κ (~11%). Casein can be prepared by isoelectric precipitation or enzyme precipitation; its composition and functional properties depend on the method used. Caseins have relatively random and flexible structures in solution; some regions contain hydrophobic residues, and others contain polar and charged residues, and thus casein tends to form micelles in solution. Casein micelles (~100–300 nm in diameter) consist of sub-micelles (10–20 nm) aggregated together. Caseins are widely used as emulsifiers, thickeners and gelling agents in various food products [14,18,19].

Whey protein is a mixture of globular proteins containing β-lactoglobulin (~55%), α-lactalbumin (~24%), serum albumin (~5%) and immunoglobulins (~15%) and constitutes the remaining ~20% of milk protein [19]. Its emulsifying properties are strongly affected by pH, ionic strength, and temperature. To prepare stable whey protein emulsions, the pH must remain sufficiently far from the isoelectric point of the protein such that it can be stabilized through electrostatic repulsion [20,21]. Egg albumin, bovine serum albumin, and human serum albumin are other widely used protein-based biosurfactants of animal origin [22].

Other well-known and physiologically important animal-derived surfactants are bile acids and pulmonary surfactants. Pulmonary surfactant (PS) is a complex mixture of lipids and proteins that coats the interior surface of the vertebrate lung as a film. It consists of about 90% lipids (mainly phospholipids) and 8–10% protein. This proteolipidic material is synthesized by type II pneumocytes and follows a regulated exocytic pathway leading to secretion into the thin aqueous layer covering the alveoli. PS exists not only in alveoli but also in bronchioles and small airways. The composition of PS varies from one species to another, and even among individuals within the same species. Although phospholipids are the main surface-active components of PS, they require the participation of the proteins to function biophysically.

The main function of PS is to maintain normal respiratory mechanics by reducing the surface tension at the alveolar air-liquid interface of lungs to avoid alveolar collapse at the end of expiration. The lack, deficiency or inactivation of PS causes severe respiratory disorders that can be lethal. Exogenous surfactant replacement therapy using either synthetic or modified natural PS extracted from bovine or porcine sources has been used to treat respiratory distress syndrome (RDS). Preclinical animal experiments and clinical practice suggest that animal-derived surfactants are superior to synthetic preparations. Difficulties in large-scale production, related to the high cost, suspension techniques, reproducibility and purity of natural surfactants, limit their clinical applications [23,24].

Challenges in the production and utilization of animal-derived surfactants comprise the high cost of animal feedstock, variations in their emulsifying properties from batch to batch, customer concerns over BSE, religious restrictions, and strict government regulations. More plant-derived surfactants are expected to replace animal-derived surfactants.

### 2.3. Plant-Derived Biosurfactants

Many surface-active compounds are derived from renewable plant resources. The European surfactant market in 2004 is estimated at 2.5 M metric tons, 25% of which are plant-derived. Here we focus on several important plant-derived surfactants that are widely used in industry or in bubble-related research and application.

Saponins are a structurally diverse class of compounds widely distributed across the plant kingdom. They can be isolated from different plant parts (e.g., roots, stems, bark, leaves, seeds, and fruits). The most significant sources of dietary saponins are the legumes: soybeans, chickpeas, mung beans, peanuts, broad beans, kidney beans and lentils; the saponin content in soybeans is 5–6% [25]. Saponins are amphiphilic molecules in which sugars are linked to either a sterol or a triterpene non-polar group, by which they are classified. Saponins have emulsifying and foaming properties, pharmacological and medicinal properties, and antimicrobial and insecticidal activity and are used in beverages, confectionery, cosmetics and pharmaceutical products [25,26].

As described above, lecithin is an important low-molecular-weight natural surfactant found in both animals and plants. Although lecithin can be extracted from animal sources, this process is too expensive for industrial applications. Instead, lecithin is predominantly manufactured from soybean oilseeds due to their abundance and low cost. Soy lecithin differs from egg lecithin in its phospholipid and fatty acid composition. The major phospholipids for soy lecithin are PC (29–46%), PE (21–34%) and phosphatidylinositol (PI, 13–21%). The concentration of total unsaturated fatty acids is much higher in soy lecithin than in egg lecithin; the egg variety contains less linoleic and nearly no linolenic acid but more long-chain polyunsaturated fatty acids than soy lecithin, which consists mainly of linoleic acid and a low amount of linolenic acid [15,16]. Various methods have been employed to improve the solubility and modify the functionality of lecithin. Soy lecithin and modified soy lecithin are widely used as emulsifiers, antioxidants, stabilizers, lubricants, wetting agents, and nutritional supplements [6].

A variety of plants produce surface-active proteins. Soy protein is one of the most important plant-derived protein surfactants. Soybeans contain about 40% protein and 20% oil. Soy proteins are mainly globulins and can be classified into 2S, 7S, 11S, and 15S fractions. Soy proteins are available in three major forms that vary in protein content: soy flours, soy protein concentrates and soy protein isolates [5,27]. Soy proteins have been used as nutritional and functional ingredients in every food category. They offer nutritional value and also affect the quality of food products. Because their properties as a surfactant are governed by factors such as solubility, hydrophobicity, molecular size and flexibility, and surface charge [28], many efforts have been devoted to improving the functional properties of soy proteins through chemical, physical and enzymatic modification.

The current increasing pressure on synthetic surfactants has stimulated the production of surfactants from renewable plant sources. Soybean oil is second largest source of vegetable oil and the second most consumed edible oil in the world. The growth in soybean oil production and the decline in dietary oil consumption due to health concerns have accelerated the development of non-food applications of soybean oil. Epoxidation is commonly used to modify soybean oil by converting its double bonds into more reactive epoxide or oxirane ring groups. The resultant epoxidized soybean oil (ESO) is a promising intermediate for the production of soybean oil–based surfactants. For example, it can be used to produce polyol surfactants through ring-opening hydrolysis [29] and polysoap surfactants through ring-opening polymerization [30,31]. The surface activity of polysoap surfactants is comparable to the reported activity of microbially derived surfactants and higher than those of some conventional synthetic surfactants; they can reduce the surface tension of Milli-Q water to a minimum value less than 30 mN/m [30,32]. The polysoap surfactant Palozengs (R-004) exhibits a unique aggregation behavior, forming small aggregates (pre-micelles) at very low concentrations [30,32]. By changing polymerization and hydrolysis conditions, novel soybean oil–based surfactants with variable structure and functionality can be produced [29,30]. With increasing health and environmental concerns over organic solvents, soybean oil–derived surfactants have been prepared using supercritical CO_2_, a more environmentally friendly solvent. Recent work [33] has revealed that polymeric surfactants possess advantageous properties over those obtained by other methods. Previous work on nanoparticle drug delivery systems and biological hydrogels prepared with soybean oil-derived polysoap surfactants [34,35] have suggested that soybean oil–derived surfactants have potential in a variety of applications due to their high surface activity, biodegradability, biocompatibility, and low cost.

## 3. Microbubble Technology and Its Applications

Microbubble technology is currently used in various industries and is expected to offer a broad range of applications, some of which are reviewed below. Ultrasound imaging is a versatile, non-invasive, low-risk, and cost-effective diagnostic modality [36,37]. Extensive studies have been performed on the use of microbubbles as ultrasound contrast agents. These microbubbles are usually encapsulated in a shell of surfactant, protein, lipid, or polymer to increase *in vivo* stability [38]. Over the past few years, special interest has been directed at the development of molecular imaging with site-targeted microbubble contrast agents, accomplished either by manipulating the chemical properties of the microbubble shell or through conjugation of disease-specific ligands of the target molecule to the microbubble surface [37]. Because the microbubbles are selectively retained in diseased tissue, this method provides a sensitive and specific diagnostic approach for the early detection of disease and analysis of disease progression [37,38]. Targeted contrast ultrasound, therefore, is expected to be used in the diagnosis of such diverse diseases as atherosclerosis, transplant rejection and tumor-related angiogenesis [37]. Ultrasound destruction of drug/gene-loaded microbubbles not only results in local release of the drugs/genes but also enhances cell membrane permeability, improving the efficiency of drug/gene delivery and making targeted contrast ultrasound an attractive therapeutic approach [39].

Amid growing environmental concerns and stricter restrictions, the potential of microbubble technology for water purification and sewage treatment is being investigated. Ozone is a powerful oxidant and is widely used in the treatment of water and wastewater. The application of ozone is limited by low solubility and poor stability in water. The effectiveness of ozonation can be improved by using ozone microbubbles. Chu *et al*. [40] examined the feasibility of using microbubble technology to treat wastewater containing a widely used non-biodegradable azo dye. Compared with a conventional bubble contactor, the microbubble system displayed a higher total mass transfer coefficient, faster decolorization, and improved total organic carbon removal efficiency. More hydroxyl radicals were produced in the microbubble system and contributed to the degradation of the dye molecules.

Gas-to-liquid mass transfer is the rate-limiting step in aerobic fermentations. A common approach in stirred tanks is to increase bubble breakup and thereby increase the interfacial area available for mass transfer by increasing the agitator’s power-to-volume ratio [41]. This approach is not economical at large scales due to increased energy costs. Because microbubbles can offer orders of magnitude more interfacial area than conventional bubbles, microbubble dispersions have been used to enhance gas-to-liquid mass transfer rates in synthesis gas fermentations. Compared to conventional sparging, a several-fold increase in the productivity of synthesis gas bioprocesses was achieved using microbubble sparging. Evaluation using a non-steady-state mathematical model indicated that mass transfer becomes more efficient as the microbubble shrinks, due to the increase in internal pressure and the liquid-phase concentration gradient at the surface of the bubble [41]. In addition to aerobic fermentation, oxygen supplied in the form of microbubbles can also be used in other oxygen-consuming processes (e.g., aquaculture, hydroponic cultivation, and aerobic treatment of sewage) [42,43]. Microbubble technology has found applications in enzyme extraction, protein recovery, bacterial harvest [44], and oil removal or recovery [9].

## 4. Properties of Some Biosurfactant-Stabilized Microbubbles

Although promising as a cost-effective and environmentally friendly technology, the lack of well-established generation and characterization methods and the lack of understanding of microbubble properties limit the application of microbubble technology. Xu *et al*. [8] revealed that the properties of microbubbles depend on their generation method and the surfactant used. Therefore, selection of a suitable method and suitable surfactant is important for the application of microbubble technology. The chemical diversity of biosurfactants provides a wide range of choices to meet specific applications. Several characterization studies have been carried out on biosurfactant-stabilized microbubble dispersions. These studies are helpful in understanding the properties of biosurfactantstabilized microbubble dispersions and are useful for their future applications. A brief review is presented below.

Feng *et al*. [45] investigated the concentration effects of the microbially derived surfactant rhamnolipid on the stability of microbubble dispersions with a mean diameter of 61–71 μm. The viscosity of the solution increases with rhamnolipid concentration. Microbubble stability increases with surfactant concentration due to increases in the viscosity of the solution, the viscoelasticity and mechanical strength of the interfacial film, and electrostatic repulsion. The effect of surfactant concentration is more pronounced at higher pH than at lower pH. Because the anionization of rhamnolipid (pKa 5.6) is principally due to the dissociation of its carboxyl head group, higher pH results in greater ionization of the surfactant and an increase in the electrostatic repulsion between adjacent ionized carboxyls. As a result, the rhamnolipid concentration at the bubble surface was reduced and the stability of the bubble dispersions was decreased. At pH 6, the dispersion stability decreased with the concentration of sodium chloride, while no effect of salt concentration on dispersion stability was observed at higher pH. At pH 6, the electrostatic repulsion between adjacent bubbles was reduced by the presence of salt, while at higher pH the salt effect was counteracted by the higher ionization degree of rhamnolipid. It is proposed that the liquid drainage of the microbubble dispersions occurs in three distinct phases. Initially, the drainage rate increases with time, due to a combination of upflow migration of bubbles and downward liquid drainage under gravity. The drainage rate then decreases with time, dominated by liquid flow under gravity. The dispersion behavior at this phase is similar to conventional wet foam. In the third phase, the drainage rate is small due to slow liquid release from films under capillarity suction. The dispersion behavior at this phase is similar to dry foam [45].

Pattle [46] characterized microbubbles prepared with lung extracts and found that the bubbles initially shrank but then maintained a constant size (12 μm). The microbubbles remained unbroken even after several washes with distilled water but disappeared in air-free water. This indicated that the bubbles were stabilized by a layer of solid, water-insoluble substance (later identified as PS) and freely permeable to air. Apparently, the stability of microbubbles is not due to an air-impermeable layer. The surface tension of the microbubbles is reportedly must be reduced to near zero, otherwise the bubbles would shrink and rapidly disappear due to increased internal pressure. The anionic surfactant Teepol, which normally leads to the displacement of other surface-active substances (e.g., saponin and albumen), did not cause the displacement of the PS. However, the adsorption of PS was inhibited by the nonionic surfactant Tween 80. The PS can be desorbed from and readsorbed to the surface without a change in its properties [46,47].

Kommalapati *et al*. [48] used a plant-derived biosurfactant containing saponin, obtained from the fruit pericarps of *Sapindus mukorossi*, to prepare microbubble dispersion. The results showed that the 10th and 50th percentile sizes are similar to those of commercial non-ionic surfactants and smaller than those of commercial ionic surfactants, while the 90th percentile size is larger than both ionic and non-ionic surfactants. The microbubble dispersion prepared with a surfactant concentration below the critical micelle concentration (CMC) had a broader size distribution than those prepared with surfactant concentrations above the CMC. The addition of salt had no effect on the size of microbubbles, probably due to the non-ionic nature of the surfactant.

Xu *et al*. [32] examined the ability of a soybean oil-derived polysoap surfactant (R-004) to form and stabilize microbubbles. The aggregation of R-004 took place at concentrations lower than the CMC determined from the inflection point of the surface tension versus concentration curve, indicating that the CMC obtained by this method may not accurately represent the concentration at which aggregation begins. A similar phenomenon was also observed in the fungi-derived protein surfactant SC3 hydrophobin [49], and the inflection point in the plot of surface tension versus concentration was thus defined as the surface-saturated concentration (SSC) rather than the CMC or CAC (critical aggregation concentration) [50]. The microbubble dispersions prepared with R-004 separated into two distinct layers within minutes of preparation: a milky upper layer and a clear lower layer. The upper layer contained larger microbubbles with an average diameter of several tens of micrometers, and the bubble size increased with time. The height of the upper layer decreased with time and finally disappeared. On the other hand, the clear lower layer contained tiny stable bubbles; most were less than 10 μm, a size that is usually difficult to prepare and even more difficult to stabilize with conventional surfactants. The formation and stabilization of microbubbles were affected by the characteristics of R- 004 aggregates. An examination of the effect of R-004 concentration indicated that the soybean oil-derived surfactant is effective in the formation and stabilization of microbubbles. R-004 likely forms a stable surface layer to provide mechanical strength, steric and electrostatic stabilization to the microbubbles. Further work is needed to elucidate the relationship between the structure and function of R-004.

## 5. Applications of Biosurfactant-Stabilized Microbubbles

Currently, applications of biosurfactant-based microbubbles are focused mainly on ultrasound diagnosis and therapy and remediation or bio-remediation of contaminants.

For decades, extensive research has been devoted to the use of microbubbles as ultrasound contrast agents for ultrasound diagnosis and therapy. These microbubbles can be stabilized by a surfactant, protein, lipid, polymer or a combination of these. The most commonly used microbubble contrast agents are albumin microbubbles. Their advantages include fragility when exposed to moderate energy ultrasound and ease of preparation. A wide range of substances, such as drugs, DNA, and virus particles, can be bound to the shells of the microbubbles, making them potential delivery systems for drugs and genes. Ultrasound-induced microbubble destruction thus provides a promising therapeutic approach for targeted treatments [51–53].

As a wide variety of organic compounds and heavy metals are released into the environment by domestic and industrial effluents, the development of efficient and cost-effective remediation methods is required. Numerous studies have demonstrated that surfactant-enhanced remediation is an effective method of treating a variety of contaminants. Surfactants are used to mobilize contaminants, readying them for remediation. They also promote the solubilization of water-insoluble contaminants by partitioning them into the hydrophobic core of micelles or lamellar structures at concentrations above the CMC. Ionic surfactants can be used to extract heavy metals through ion exchange, precipitation-dissolution, and counter-ion binding [54]. However, the application of surfactant-enhanced remediation is hindered by the possibility of spreading of the contaminated zone and further contaminating ground water. The combination of foam technology and biosurfactants is a solution that has recently received increasing attention. Rhamnolipid foam consisting of many tiny bubbles exhibits a higher efficiency in the removal of heavy metals compared with distilled water and surfactant solution [55]. It was postulated that the metals were removed through the formation of complexes with the surfactants on the soil surface, which detached them from the soil and brought them into solution. Anionic biosurfactants are effective in the removal of cationic metals owing to their high surface activity and the electrostatic metal—surfactant interaction. High pH conditions are likely to give better outcomes as a result of enhanced metal solubility and surfactant activity.

Ripley *et al*. [56] described the development of a protein-based foam as a carrier system for the delivery of microbes, nutrients, and oxygen to treat hydrocarbon-contaminated soil. Foam stability increased with the concentration of protein hydrolysate. The addition of metal salts at relatively low concentrations greatly increased the quality and stability of the foam. Although the addition of viscosity modifiers (sodium alginate, carboxymethyl cellulose, and xanthan gum) retarded drainage, it has deleterious effects on foam generation and oxygen transfer. The inhibition of oxygen transfer due to increasing viscosity may result in anoxic conditions. Moreover, these viscosity modifiers may be consumed by the microbes as an alternative carbon source and may thus prevent or delay the biodegradation of hydrocarbon. In situ generation of the bioactive foam is preferred to achieve high treatment efficiency. Compared to the non-foamed controls, enhanced *n*-hexadecane degradation was observed. Furthermore, 32.9% of the *n*-hexadecane was degraded in the columns treated with aerated foam, and 51.3% was degraded in the columns treated with oxygenated foam, indicating that oxygen-supplemented bioactive foam is more effective than aerated bioactive foam in the removal of *n*-hexadecane from the soil columns. The dissolved oxygen concentration in the foams generated with oxygen remained significantly higher than in the foams generated with air, supporting the hypothesis that biodegradation was enhanced by the sustained release of rate-limiting oxygen onto the *n*-hexadecane-contaminated soil and the associated improved oxygen transfer [57].

Microscopic observation has revealed bubbles densely covered with bacterial cells and significantly fewer bacteria in the aqueous phase of the foam lamellae, demonstrating the preferential sorption of the degrader at the bubble surface. The retention of bacteria within the foam is correlated to the cell surface hydrophobicity of the organisms used. Generally, proteins unfold and rearrange when they adsorb at the surface and expose their hydrophobic groups to the air phase. This process may facilitate the sorption of hydrophobic bacterial cells to the surfaces of bubbles. The protein hydrolysate used not only acts as a surfactant but also as a nitrogen-rich nutrient source. These results suggest the potential of protein-based bioactive foams for the bioremediation of contaminated sites [57].

Kommalapati *et al*. [58] demonstrated that microbubble dispersions prepared with the plant-derived surfactant described above can be used to remove hydrophobic organic compounds (HOCs) from soil, using hexachlorobenzene (HCB) as the model HOC. The recovery of HCB from soil columns using the biosurfactant-prepared microbubble dispersions was considerably higher than a simple waterflood. The recovery of HCB increased with surfactant concentration due to increased solubility of HCB.

Choi *et al*. investigated the use of a saponin-based microbubble suspension to enhance aerobic biodegradation of phenanthrene in a sand column [59]. The gas content of the microbubble dispersion prepared with 2% saponin was about 40% [60]. The addition of salt did not affect the properties of the microbubble dispersions, and the delivery of oxygen and phenanthrene-degrading bacteria were confirmed. Compared with saponin solution, the biodegradation of phenanthrene was improved; this effect could be further enhanced by repeated introduction of microbubble dispersions. The results revealed that microbubble dispersions prepared with plant-derived surfactants can be used as potential carriers of oxygen, pollutant-degrading microorganisms, and micronutrients to enhance aerobic biodegradation under oxygen-limiting environments.

## 6. Conclusions

Increasing concern over the supply, price and environmental impact of petrochemical surfactants has resulted in increased demand for surfactants derived from natural sources, especially plants. Much more work on the characterization of biosurfactants remains to be done to fully exploit their properties. As the production of vegetable oil grows and dietary oil consumption declines, the production of vegetable oil–based surfactants is expected to increase. Further study of the controlled production and characterization of these surfactants is needed. Compared to biosurfactant-stabilized emulsions, knowledge of biosurfactant-stabilized microbubble dispersions is insufficient, which limits their application. Investigation into the effects of factors such as pH, ionic strength, and temperature on microbubbles will be helpful for understanding their formation and stabilization. As nanobubbles gain interest from both fundamental and applied points of view, the preparation and stabilization of nano-sized bubbles with biosurfactants remains a major challenge.
